# Lentiviral vector-mediated *RBM5* overexpression downregulates *EGFR* expression in human non-small cell lung cancer cells

**DOI:** 10.1186/1477-7819-12-367

**Published:** 2014-12-02

**Authors:** Zhenzhong Su, Jinzhi Yin, Lijing Zhao, Ranwei Li, Hong Liang, Jie Zhang, Ke Wang

**Affiliations:** Department of Respiratory Medicine, The Second Affiliated Hospital of Jilin University, No.218 Ziqiang Street, Nanguan District, Changchun, Jilin 130041 China; Department of Pathophysiology, Norman Bethune College of Medicine of Jilin University, No.126 Xinmin Street, Chaoyang District, Changchun, Jilin 130021 China; Department of Urinary Surgery, The Second Affiliated Hospital of Jilin University, No.218 Ziqiang Street, Nanguan District, Changchun, Jilin 130041 China; Department of Respiratory Medicine, Changchun General Hospital, No.1810 Renmin Street, Nanguan District, Changchun, Jilin 130021 China

**Keywords:** RNA binding motif 5, Epidermal growth factor receptor, Non-small cell lung cancer, Lentiviral vector, A549, Xenograft mice model

## Abstract

**Background:**

RNA binding motif 5 (*RBM5*) is a tumor suppressor gene that modulates apoptosis through the regulation of alternative splicing of apoptosis-related genes. Our previous studies suggested that *RBM5* expression was negatively correlated with the expression of epidermal growth factor receptor (*EGFR*) in non-small cell lung cancer (NSCLC) tissues. This study was aimed at determining whether *RBM5* is able to regulate *EGFR* expression.

**Methods:**

Both *in vitro* and *in vivo* studies were carried out to determine the effect of *RBM5* on the expression of *EGFR*. Lentiviral vector-mediated *RBM5* overexpression was employed in lung adenocarcinoma cell line A549. A549 xenograft mice were treated with recombinant *RBM5* plasmid carried by attenuated *Salmonella* typhi Ty21a. Real-time quantitative polymerase chain reaction and Western blot were carried out to detect *RBM5* and *EGFR* expression.

**Results:**

Both *in vivo* and *in vitro* studies indicated that the expression of *EGFR* mRNA and protein was decreased significantly in the *RBM5* overexpression group compared to control groups as shown by real-time PCR and Western blot analysis. We identified that *RBM5* overexpression inhibited *EGFR* expression both in A549 cells and in A549 xenograft mice model.

**Conclusions:**

Our study demonstrated that *EGFR* expression is regulated by *RBM5* in lung adenocarcinomas cells either in a direct or indirect way, which might be meaningful with regards to target therapy in lung cancer.

## Background

Lung cancer is one of the most common malignant tumors and remains the leading cause of cancer death both in males and females globally [1]. Among all lung cancer subtypes, non-small cell lung cancer (NSCLC) accounts for approximately 87% of all lung cancer cases, and has a poor prognosis; the overall five-year survival rate is 18.2% [2]. Molecularly, NSCLC development is believed to be initiated by the activation of oncogenes or inactivation of tumor suppressor genes [3].

Epidermal growth factor receptor (*EGFR*) (also known as *HER-1* or *Erb1*) is a cell-surface receptor belonging to the *ErbB* tyrosine kinase receptor family, which also includes *HER-2/neu* (*ErbB2*), *HER-3 (ErbB3)*, and *HER-4 (ErbB4). EGFR* activation is associated with cell apoptosis, proliferation, angiogenesis, invasion, and metastasis, which plays an important role in carcinogenesis and tumor progression in human epithelial cancers, including NSCLC [4]. These actions are accomplished through activation of the *RAS-RAF-MEK-ERK* and *PI3K-AKT-mTOR* pathways [5]. *EGFR* and PI3K initiate malignant neoplastic transformation via a combinatorial genetic network composed of other pathways, including the *Tor*, *Myc*, *G1 Cyclins-Cdks*, and *Rb-E2F* pathways [6], and drive cells through the restriction point of late G(1) into S phase [7]. A series of anticancer agents directly targeting *EGFR* were developed and proved to be effective [8–13], but the clinical benefits of *EGFR*-TKIs are limited by primary or acquired resistance [14]. Therefore, *EGFR* inhibition by an upper regulator seems to be more attractive. Yet the *EGFR* upstream regulatory mechanisms are still not well understood. Further insights into important molecular regulators of *EGFR* are needed for the development of novel therapeutics*.*

RNA-binding motif protein 5 (*RBM5*) (also known as *LUCA-15* or *H37*) maps to the human chromosomal locus 3p21.3, which is strongly associated with lung cancer [15]. It is reported to be downregulated in 73% of primary NSCLC specimens [16] and is also found in other human cancers. However, the precise mechanism by which *RBM5* mediated tumor suppression still remains to be clarified. Present studies are mostly focused on the regulation of apoptosis by the alternative splicing of correlated genes, such as *Bax, Bcl-2,* cleaved *caspase-3, caspase-9*, and *P53* [17–21]. Only a few researchers noticed another mechanism of negative regulation of cell proliferation, inducing cell cycle arrest in G1 by downregulating *cyclin A* and phosphorylated *RB* expression [17], which might also be involved in the malignant neoplastic transformation initiated by the *EGFR* and *PI3K* signaling pathway. These observations draw our interest in regard to the relationship between *RBM5* and *EGFR*. We conducted a series of investigations to clarify the relationship between *RBM5* and an important regulator of cell proliferation, *EGFR*. We detected *RBM5* and *EGFR* expression in 120 paired resected NSCLC tumor tissues and adjacent normal tissues in a previous study, which suggested that the *RBM5* expression was negatively correlated with the expression of *EGFR* in NSCLC tissues [22]. Afterwards, we inhibited *EGFR* expression in the lung adenocarcinoma cell line NCI-H1975 using small interfering RNA, and found that *RBM5* expression was not directly regulated by *EGFR* in non-smoker-related lung adenocarcinomas [23]. Herein, we hypothesized that inhibition of *EGFR* in lung adenocarcinomas might be achieved via *RBM5* overexpression. The objective of this study was to assess whether forced *RBM5* expression in lung adenocarcinoma cell line A549 cells and A549 xenografts could suppress the expression of *EGFR*, which would suggest that one of the mechanisms of potential tumor suppressor activity of *RBM5* in NSCLC is initiated via the inactivation or inhibition of *EGFR*.

## Methods

### Cell culture

Human lung adenocarcinoma cell line A549 cells were purchased from the Chinese Academy of Medical Sciences (Beijing, China). Cells were grown in Roswell Park Memorial Institute (RPMI) 1640 supplemented with 10% fetal bovine serum (Gibco, Grand Island, United States), and maintained at 37°C in a humidified 5% CO_2_ atmosphere.

### Lentiviral vectors construction and lentivirus infection

Lentiviral vectors containing green fluorescence protein (*GFP*) were employed in order to achieve high efficiency of introduction and subsequent stable expression of RBM5 in A549 cells. Recombined *pGC-LV-GV287-GFP* vector with the *RBM5* (NM_005778) gene (*LV-RBM5*) and *pGC-LV-GV287-GFP* with a scrambled control sequence (*LV-GV287*) were constructed by Genechem Company (Genechem, Shanghai, China). A549 cells were then infected with the above lentiviral vectors. A total of 5 × 10^5^ A549 cells were seeded in a six-well cell plate and further incubated for 12 hours to reach 30% confluent, and then infected with *LV-RBM5* (*RBM5* overexpression group), *LV-GV287* (negative control group), and no infection (non-transfected control group) by replacing the infection medium containing recombinant vectors at a multiplicity of infection (MOI) of 20 plaque-forming units (p.f.u.) per cell. Plates were then incubated for 24 hours prior to having their media changed to fresh, virus-free media. Three days later, the *GFP* density contained by lentivirus was detected to evaluate the efficiency of infection, and cells were harvested for Western blot and real-time quantitative polymerase chain reaction (RT-qPCR) analysis.

### Establishment of A549 xenografts

The use of animals in this study was in accordance with animal care guidelines, and the protocol was approved by Jilin University Animal Care Committee. A549 xenografts were established and the *RBM5* gene was delivered into xenografts by attenuated *Salmonella* according to a previous study [19]. Briefly, BALB/c athymic nude female mice (nu/nu); between four and five-weeks-old) were purchased from the Institute of Zoology, Chinese Academy of Sciences (Beijing, China). A549 cells (1 × 10^7^) were suspended in 100 μl PBS and injected subcutaneously into the right flank region of nude mice.

Competent *Salmonella enterica* serovar typhimurium cells (competence) (obtained from the China-Japan Union Hospital of Jilin University, Jilin, China) were mixed with 1 μg *GV287*-*RBM5* or 1 μg *GV287* plasmids and cooled for 15 minutes on ice. The plasmids were electro-transfected into the competence under the conditions as follows: capacitance = 25 μF, voltage = 1.25 kV (12.5 kV/cm). Then the recombinant attenuated salmonellae were quickly transferred into Luria-Bertani (LB) agar medium for proliferation at 37°C and stored at −80°C.

The tumor-bearing mice were randomly divided into three groups (six mice per group) at day 21 after cell injection. The mice were treated at day 28 and 35, respectively, through a tail vein as follows: (a) control group (50 μl PBS); (b) negative control group (attenuated *Salmonella*-carrying *GV287*) (10^8^ colony-forming units (CFU) per 50 μl PBS); (c) *RBM5* overexpression group (attenuated *Salmonella*-carrying *GV287-RBM5*) (10^8^ CFU per 50 μl PBS). The mice were sacrificed on day 42 and the tumors were removed. One part of the tumor was fixed in Trizol™ reagent (Invitrogen, Carlsbad, United States) for RT-qPCR, and another part was immediately snap-frozen in liquid nitrogen for Western blot analysis.

### Protein extraction and Western blot

Total protein from both tumor tissues and cultured cells was extracted according to a previous study [22]. Briefly, protein concentration was measured by the Protein Assay Kit (Bio-Rad Laboratories, Richmond, United States). Equal amounts of protein samples (30 μg) were separated by 8% SDS-PAGE and transferred onto poly (vinylidene fluoride) (PVDF) membranes (Millipore, Boston, United States). The membranes were treated with tris-buffered saline and Tween-20 solution (Sigma, California, United State) (TBST) containing 50 g/L skimmed milk at room temperature for one hour, and incubated overnight at 4°C with a monoclonal antibody against *RBM5* (Santa Cruz Biotechnology, California, United States) or *EGFR* (Proteintech Group, Chicago, United States).The mouse monoclonal antibody against *β-actin* (Proteintech Group, Chicago, United States) was used as a housekeeping control gene. Membranes were washed three times for 10 minutes with TBST and incubated with horseradish peroxidase-conjugated secondary antibodies (Proteintech Group, Chicago, United States) at a dilution of 1:500 for one hour at room temperature. Membranes were washed three times for 10 minutes with TBST, and bands were detected using an Amersham ECL Plus Western Blotting Detection Reagents (General Electric Company, Fairfield, United States).The protein levels were quantified by densitometry using Quantity One software (Bio-Rad Laboratories, Richmond, United States).

### RNA extraction and real-time quantitative polymerase chain reaction

Total RNA was extracted using Trizol reagent (Invitrogen, California, United States) according to the manufacturer’s instructions. The ratio of absorbance at 260 and 280 nm (A260/280) was used to assess RNA purity and quantity. First-strand cDNA was generated using M-MLV Reverse Transcriptase (Promega, Madison, United States) and Oligo(dT) primers (Sangon Biotech, Shanghai, China) according to the manufacturer’s instructions. Primers were made by Genechem (Genechem, Shanghai, China). Selected primer sequences included *RBM5* forward 5′-CCATCACAGAGAGCGATATTCG-3′, *RBM5* reverse 5′-CGGCTTACACCTGTTTTCCTC -3′, *EGFR* forward 5′-ATGAGATGGAGGAAGACGG -3′, *EGFR* reverse 5′-CGGCAGGATGTGGAGAT-3′, glyceraldehyde-3-phosphate dehydrogenase (*GAPDH*) forward 5′-TGACTTCAACAGCGACACCCA-3′, and *GAPDH* reverse 5′-CACCCTGTTGCTGTAGCCAAA-3′.

RT-qPCR was carried out using a Thermal Cycler Dice Real Time System (TaKaRa, Osaka, Japan) using Prime Script™ RT Master Mix (TaKaRa, Osaka, Japan). A two-step cycling condition was used for *EGFR, RBM5*, and *GAPDH* as follows: 95°C for 30 seconds followed by 40 cycles of 95°C for five seconds, and then 60°C for 30 seconds. A dissociation curve was generated for all three genes using the following conditions: 95°C for 15 seconds, 55°C for 30 seconds, and then 95°C for 15 seconds. The expression levels of the *RBM5* and *EGFR* genes were normalized to the internal control *GAPDH*, respectively, to obtain the relative threshold cycle (ΔCt), and the relative expression between control A549 cells and infected cells was calculated using the comparative Ct (ΔΔCt) method (ΔΔCt = ΔCt of control cells – ΔCt of infected cells ) or 2-ΔΔCT.

### Statistical analysis

All experiments were performed at least in triplicate. All data were presented as means ± standard deviation (SD). Statistical significance was determined by analysis of t-test using SPSS version 17.0 (SPSS Inc., Chicago, United States). A *P* value of less than 0.05 was considered statistically significant.

## Results

### Infection efficiency of lentivirus vectors

**Figure 1:**
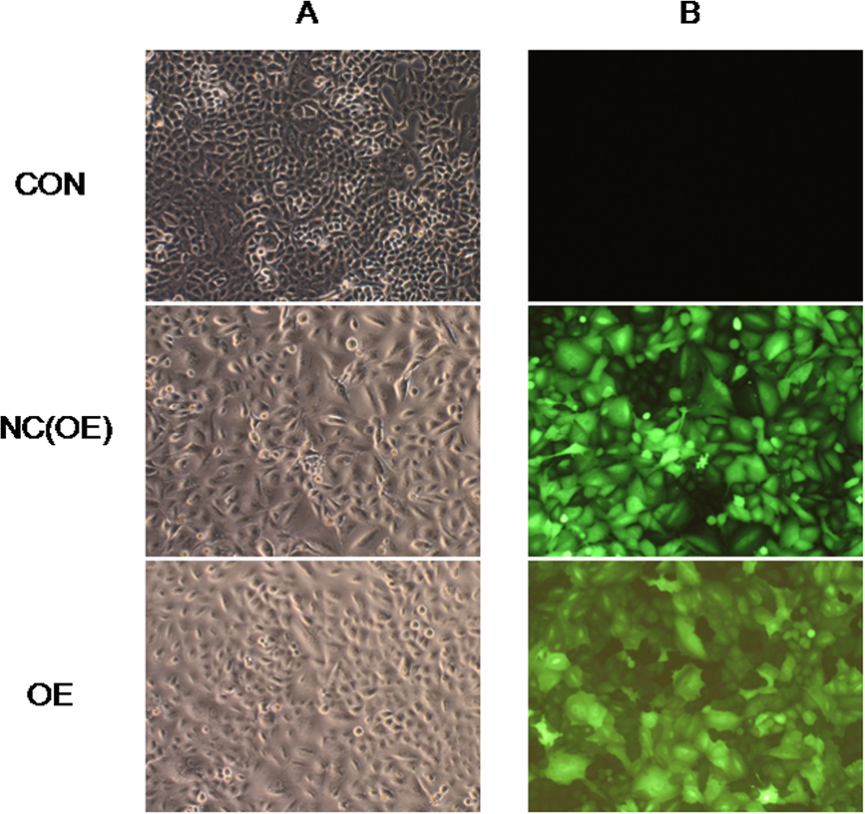
**Infection efficiency of** ***LV-RBM5*** **in A549 cells by GFP detection (100×).** Lentiviral vector-mediated *RBM5* expression was visualized by fluorescence microscopy three days after infection. Comparing the assessment in a bright field with the assessment in fluorescent field revealed an infection efficiency of over 80%. **A**, bright field; **B**, fluorescent field; CON, control group with no transfection; NC(OE), negative control group transfected with *GFP* lentiviral vectors LV-GV287; OE, *RBM5* overexpression group transfected with *GFP* lentiviral vectors LV-RBM5.

In order to achieve high efficiency of introduction and subsequent stable expression of *RBM5* in A549 cells, we tried to import this gene by infecting A549 cells with *RBM5* lentiviral vectors containing *GFP*. The recombinant lentivirus vector *LV-RBM5* was successfully constructed and infected A549 cells. The stably infected A549 cells expressed *GFP* after infection by the lentiviral vectors at different MOIs. *GFP* expression was detected three days after infection using fluorescence microscopy. The efficiency of the infection (averaged proportion of *GFP*-expressing cells on the total cell count) was approximately 80% at an MOI of 20 (Figure 1). Consequently, an MOI of 20 was chosen for the next steps of this study.

### Overexpression of *RBM5*in A549 cells infected with lentiviral vectors

**Figure 2:**
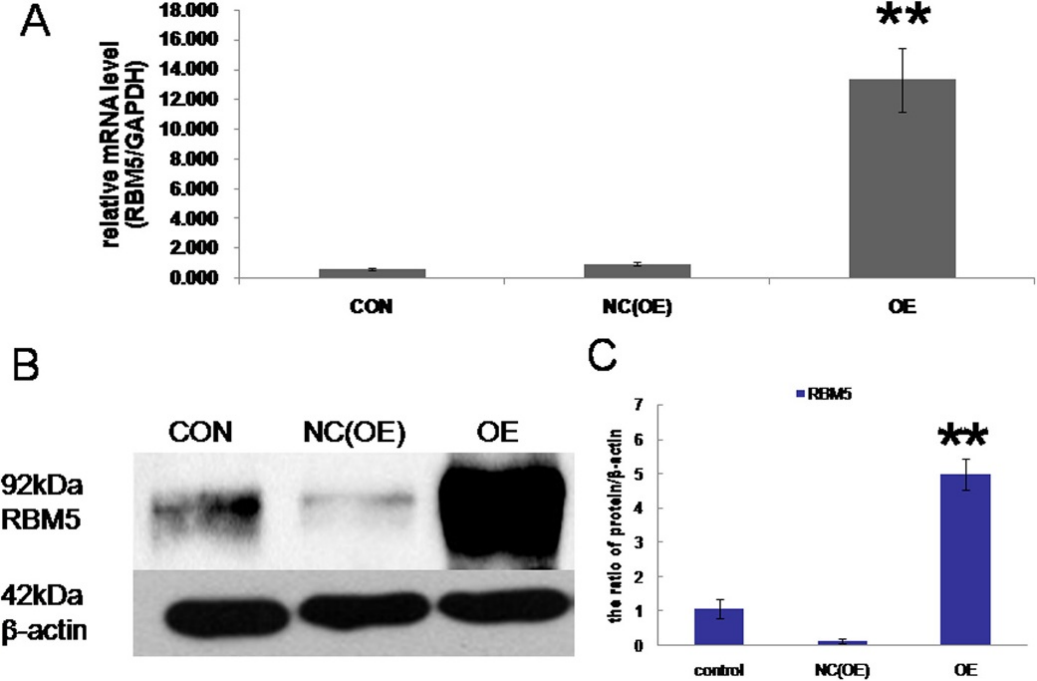
**Overexpression of** ***RBM5*** **in A549 cells by lentivirus-mediated gene expression system.** A549 cells were infected with lentiviral vector LV-*RBM5*. Three days after infection, the relative RBM5 mRNA and protein expression were determined by RT-qPCR and Western blot analysis respectively. β-actin was used as an internal control. **A**, RT-qPCR analysis for RBM5 mRNA in different groups. **B**, Western blot analysis for RBM5 protein in different groups. **C**, Quantification of RBM5 protein levels relative to β-actin; CON, non-transfected control group; NC(OE), negative control group; OE, RBM5 overexpression group. Data shown are means ± SD of three separate experiments. **P <0.01 indicates significant difference as compared to the control.

RT-qPCR analysis demonstrated that the relative expression level of *RBM5* mRNA was markedly increased in the *RBM5* overexpression group (13.32 ± 2.16), compared with that in the negative control group (1.00 ± 0.09) and the non-transfected control group (0.65 ± 0.07; *P* <0.01; Figure 2A). There was no statistical difference of *RBM5* mRNA expression between the negative control and the non-transfected control (*P* >0.05). Furthermore, the effectiveness of the lentiviral infection of *LV-RBM5* was also confirmed by Western blot analysis. The protein expression of *RBM5* in A549 cells was significantly higher in the *RBM5* overexpression group than that in the control groups (Figure 2B).

### Overexpression of *RBM5*inhibits *EGFR*expression in A549 cells

**Figure 3:**
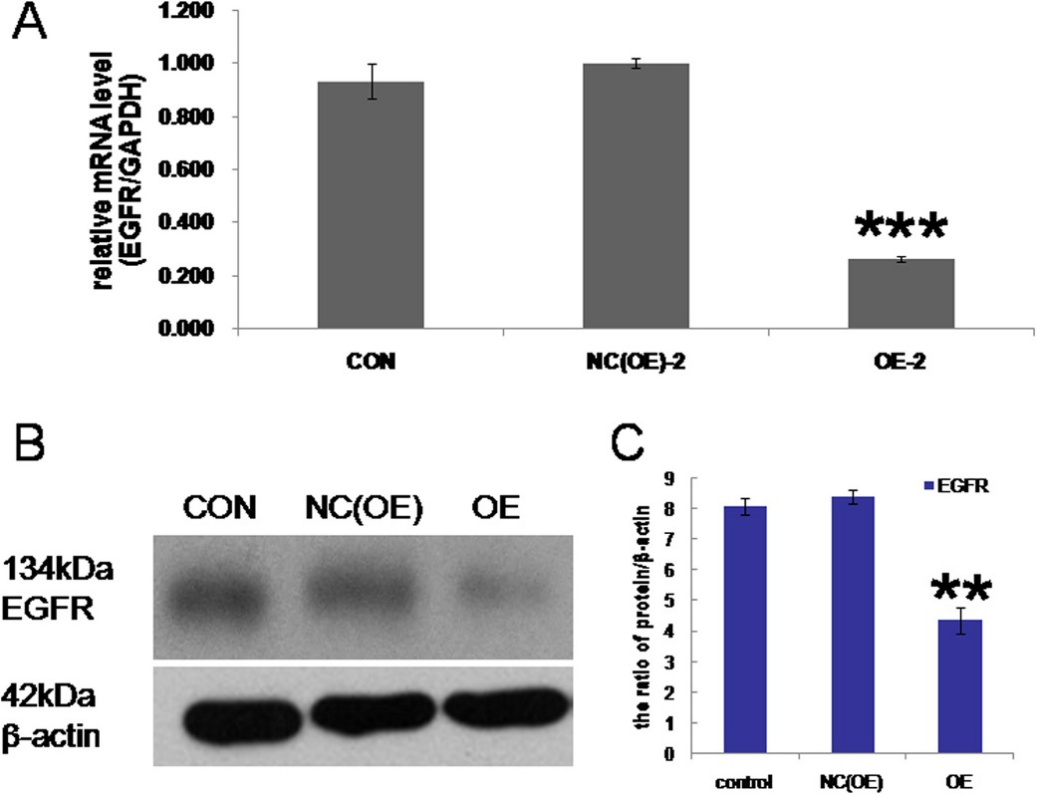
***EGFR*** **expression was suppressed by** ***RBM5*** **overexpression in A549 cells.** A549 cells were infected with lentiviral vector *LV-RBM5*. Three days after infection, the relative *EGFR* mRNA and protein expression were detected by RT-qPCR and Western blot analysis, respectively. *β-actin* was used as an internal control. **A**, RT-qPCR analysis for *EGFR* mRNA in different groups. **B**, Western blot analysis for *EGFR* protein in different groups. **C**, Quantification of *EGFR* protein levels relative to *β-actin*; CON, non-transfected control group; NC(OE), negative control group; OE, *RBM5* overexpression group. Data shown are means ± SD of three separate experiments. **P <0.01 and ***P <0.001 indicates significant difference as compared to the control.

To explore whether *RBM*5 is able to directly regulate *EGFR* expression, we examined *EGFR* mRNA by RT-qPCR, and *EGFR* protein by Western blot analysis in A549 cells infected by different lentiviral vectors. As seen in Figure 3A, compared to negative control group and non-transfected control group, cells in the *RBM5* overexpression group showed a significant decrease in *EGFR* expression (by 28.5% and 26.6%, respectively; *P* <0.001 and *P* <0.001, respectively). Additionally, Western blot analysis showed that the protein expression of *EGFR* in the *RBM5* overexpression group was significantly lower than that in the control groups (Figure 3B).

### Suppression of *EGFR*expression by *RBM5*overexpression in A549 xenograft tumors

**Figure 4:**
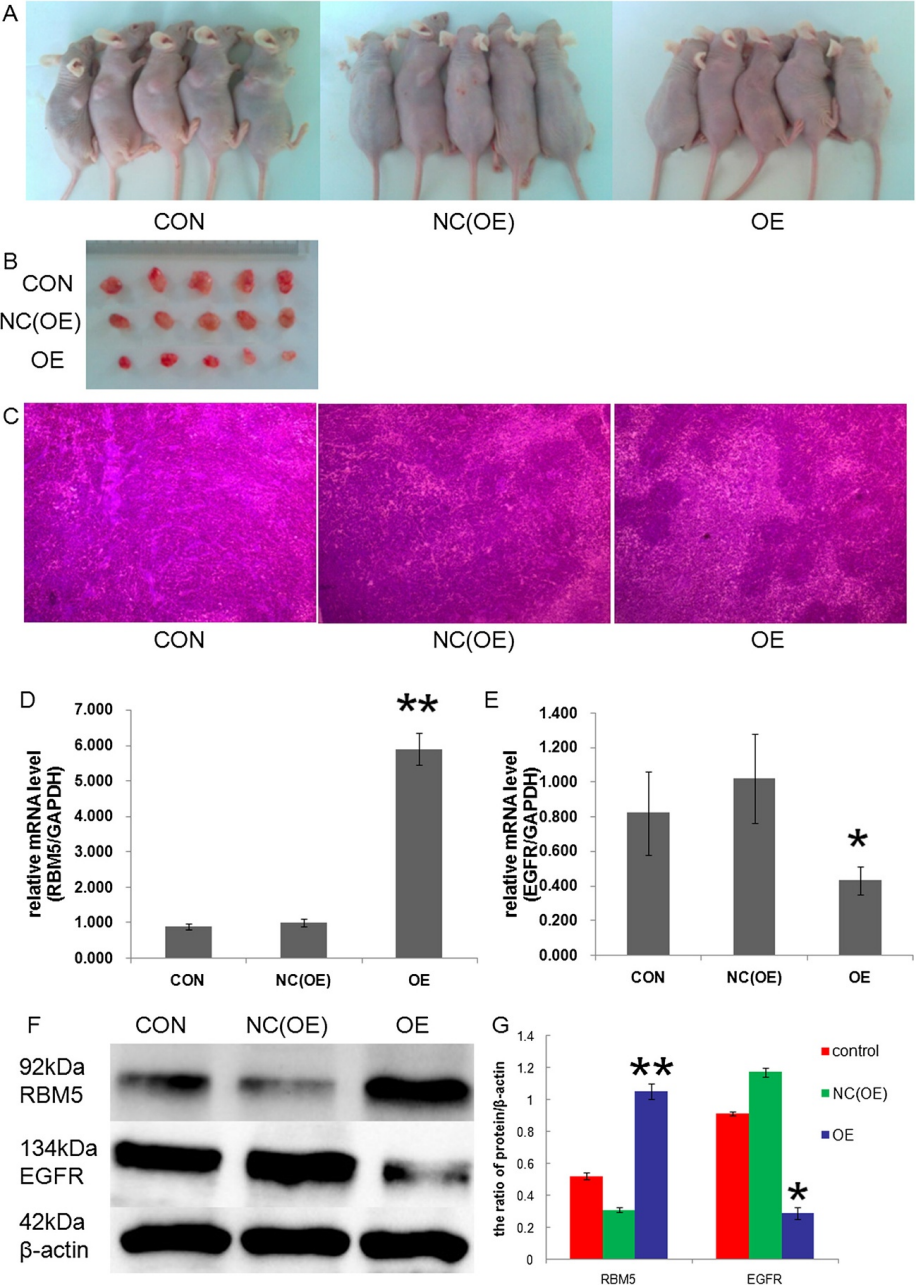
***EGFR*** **expression was suppressed by** ***RBM5*** **overexpression in A549 xenograft tumors.** A549 xenografts were established and the *RBM5* gene was delivered into xenografts by attenuated *Salmonella*. The mice were sacrificed on day 42. **(A)** Tumor sizes in nude mice in different groups. **(B)** Comparision of A549 xenografts taken out from different groups. **(C)** H&E staining of tumors of nude mice in different groups (40×). **(D)** RT-PCR analysis of the expression of relative *RBM5* mRNA in the xenograft tumors. **(E)** RT-PCR analysis of the expression of relative *EGFR* mRNA in the xenograft tumors. **(F)** Western blot analysis of *RBM5* and *EGF*R protein expression in the xenograft tumors. **(G)** Quantification of protein expression relative to *β-actin*. β-actin was used as an internal control. CON, non-transfected control group; NC(OE), negative control group; OE, *RBM5* overexpression group. Data shown are means ± SD of three separate experiments. *P <0.05 and **P <0.01 indicate significant difference as compared to the negative control.

To further test our hypothesis, the mice model of A549 xenograft was established as described in the Methods section. To ensure that the recombinant attenuated salmonellae-carrying plasmids preferentially localized in the xenograft tumors, the kinetics of bacterium distribution in the xenograft tumor and different organs of the tumor-bearing mice were monitored in a previous study [18]. At the 28th and 35th day after implantation, the tumor-bearing mice were treated with attenuated *Salmonella*-carrying plasmid through a tail vein. The mice were sacrificed 42 days after implantation and the tumors were removed to monitor the tumor sizes and to determine *RBM5* and *EGFR* expression by RT-qPCR and Western blot analysis. H&E staining were performed to observe histopathological performance on A549 xenografts. We observed that the mRNA and protein expression of *RBM5* were significantly increased in the *RBM5* overexpression group as compared to those in control groups, while *EGFR* expression was decreased significantly in the *RBM5* overexpression group as compared to those in control groups (Figure 4). H&E staining showed that there were a large number of cancer nests in control, and that the tumor tissue could survive in a good state (Figure 4).

## Discussion

The *RBM5* gene is a tumor suppressor gene (*TSG*) that is located within a 370 kb overlapping lung cancer allelic loss region on 3p21.3 [24]. There is increasing evidence suggesting that downregulation of *RBM5* plays an important role in NSCLC occurrence, progression, metastasis, and drug resistance [16, 18, 21, 22, 25, 26], yet the mechanisms are still not well clarified. Present studies on *RBM5* anti-tumor mechanisms are mostly focused on its apoptosis induction role, such as: *RBM5* overexpression enhanced *TRAIL-, TNF-alpha-, Fas-,* and *P53*-mediated apoptosis [20, 27, 28], increased the expression of *Stat5b, BMP5* [29], *Bax* [17], and *proapoptotic Casp-2 L* [30], and decreased the expression of *Amplified In Breast Cancer 1 (AIB1), proto-oncogene Pim-1, caspase antagonist BIRC3 (cIAP-2, MIHC)*, and *cyclin-dependent kinase 2 (CDK2)* [29]. *Rac1* and *β-catenin* were upregulated when *RBM5* was knocked down [26]. Our previous study confirmed previous findings and further demonstrated that exogenous expression of *RBM5* inhibited the A549 cell growth *in vivo* and *in vitro*, and re-sensitized A549/DDP cells to cisplatin by enhancement of mitochondria apoptosis [18, 19, 21]. Our recent study demonstrated for the first time an inverse correlation between the expression levels of *RBM5*, and transforming *growth factor alpha (TGF-α)* signaling factors, *EGFR*, and *KRAS* in NSCLC tissues [22], which suggested that the presence of a complex regulatory network between those genes was involved in tumor suppression and oncogenic expression. Although several studies found that the molecular mechanism of *RBM5* tumor suppression involved cell proliferation inhibition [17, 29, 31], the precise mechanisms underlying such inhibition have been poorly understood. Here, we demonstrate that overexpression of *RBM5* suppressed *EGFR* expression, both in lung adenocarcinoma cell line A549 cells and in A549 xenograft tumors. This effect occurs in NSCLC cells expressing a lower level of *RBM5* [17]. Previously, we have proved that *RBM5* expression was not directly regulated by *EGFR* [23], however, the results in the current study indicate that *RBM5* might manipulate *EGFR* expression as an upstream gene, which may be a predominant mechanism by which *RBM5* mediates tumor suppression.

Bonnal *et al*. found that *RBM5* was a component of complexes involved in 3′ splice site recognition, and regulates alternative splicing of apoptosis-related genes, including the Fas receptor, switching between isoforms with antagonistic functions in programmed cell death [28]. It may be the same mechanism that explains how *EGFR* expression was suppressed by overexpression of *RBM5*. That is, upregulated *RBM5* recognized the 3′ splice site of the pre-mRNAs of *EGFR* and led to more alternatively spliced mRNAs and less matured mRNAs of *EGFR*. However, we could not definitively conclude what the alternatively spliced mRNAs are. The alternatively spliced mRNAs might be mRNAs of other genes, generate protein isoforms of the same gene which harbor different functions, or degenerated.

For many years, chemotherapy has been the standard first-line systemic treatment for advanced NSCLC, but the clinical outcomes were unpromising. The advent of *EGFR* tyrosine kinase inhibitors (TKIs) changed the treatment paradigm. Nevertheless, the clinical application is restricted by limitations [8–14], including: (1) patients should be selected on the basis of *EGFR* mutations rather than *EGFR* amplification or overexpression; and (2) primary or acquired drug resistance after a short time of usage. Previous studies found that in tumor biopsy samples, 55 to 61% of the samples were *EGFR*-positive and 32 to 45% had *EGFR* amplification, without fully overlapping each other [32–34]. Yet, the occurrence of *EGFR* gene mutations was only 10 to 40% [35–38]. Our present findings suggest that overexpression of *RBM5* could inhibit *EGFR* expression by either direct or indirect ways in A549 cells. The cell line A549 was chosen for this study not only because it has the lowest *RBM*5 expression in seven different lung cancer cell lines [17], but also because it has wild-type *EGFR*-positive expression and gene amplification [39, 40], which are more common in NSCLC. It might be concluded that NSCLC with *EGFR*-positive expression or gene amplification could be treated by exogenous *RBM5*, resulting in *EGFR* suppression. Our results could have a potential implication for lung cancer treatment, and uncover a new promising therapeutic strategy to suppress the *EGF*R pathway, which is induced by the overexpression of *RBM5*. Taken together, our study demonstrates a prospective meaning that overexpression of *RBM5* in NSCLCs would lead to tumor suppression through *EGFR* inhibition. *RBM5* may act as a novel therapeutic target in terms of gene therapy.

Nevertheless, there were still several limitations in the present study. Firstly, as we have focused on the A549 cell line, additional experiments involving other cancer cell lines or normal and/or immortalized cell lines would help to verify this relationship between these two genes. Secondly, as the upstream regulation of *EGFR* is still not well understood, further studies concerning whether there are other mechanisms involving in this process are warranted in order to confirm the specific mechanisms of *EGFR* expression suppression. Thirdly, the relationship between *RBM5* and *EGFR* mutation is yet unknown. Further investigation is required to determine whether *RBM5* is able to modulate *EGFR* expression when *EGFR* mutations exist.

## Conclusions

We demonstrate that *EGFR* expression is regulated by *RBM5 in vivo* and *in vitro* in a direct or indirect way, and that may be one of the predominant mechanisms by which *RBM5* mediated tumor suppression. These findings also indicate that *RBM5* is the upstream regulator of the *EGFR* pathway.

## Abbreviations

RBM5: RNA binding motif 5
EGFR: epidermal growth factor receptor
NSCLC: non-small cell lung cancer
RPMI: Roswell Park Memorial Institute
GFP: green fluorescence protein
LV-RBM5: Recombined pGC-LV-GV287-GFP vector with the RBM5 (NM_005778) gene
LV-GV287: pGC-LV-GV287-GFP with a scrambled control sequence
MOI: multiplicity of infection
p.f.u.: plaque-forming units
RT-qPCR: real-time quantitative polymerase chain reaction
CFU: colony-forming units
TBST: tris-buffered saline and Tween-20 solution
GAPDH: glyceraldehyde-3-phosphate dehydrogenase
SD: standard deviation
TSG: tumor suppressor gene
TKIs: tyrosine kinase inhibitors.
